# Prognostic relevance of resection at first recurrence in isocitrate dehydrogenase mutant lower-grade glioma: results from a retrospective, single-center, volumetric analysis

**DOI:** 10.1007/s11060-025-05353-x

**Published:** 2026-03-11

**Authors:** Christine Jungk, Karla Goepfert, Mara Gluszak, Karim Marhaba, Philip Dao Trong, Sandro M. Krieg, Martin Bendszus, Felix Sahm, Andreas W. Unterberg

**Affiliations:** 1 Department of Neurosurgery, Heidelberg University Hospital, Im Neuenheimer Feld 400, D-69120 Heidelberg, Germany; 2 Department of Neurosurgery, Medical Faculty, Heidelberg University, D-69120 Heidelberg, Germany; 3 Department of Neuropathology, Heidelberg University Hospital, Im Neuenheimer Feld 224, D-69120 Heidelberg, Germany; 4 Clinical Cooperation Unit Neuropathology, German Cancer Consortium (DKTK), German Cancer Research Center, Im Neuenheimer Feld 224, D-69120 Heidelberg, Germany; 5 Department of Neurosurgery, Vivantes Klinikum Neukölln, Rudower Str. 48, D-12351 Berlin, Germany; 6 Department of Neuroradiology, Heidelberg University Hospital, Im Neuenheimer Feld 400, D-69120 Heidelberg, Germany

**Keywords:** Lower-grade glioma, IDH mutation, Repeat resection, Survival, Functional outcome, Tumor volumetry

## Abstract

**Purpose:**

The prognostic role of repeat resection in IDH-mutant lower-grade glioma remains insufficiently defined. This observational single-center study investigated whether resection at 1^st^ recurrence was associated with (progression-free) survival after recurrence (PFS-2; SAR) and evaluated functional outcomes.

**Methods:**

We retrospectively analyzed 148 molecularly characterized IDH-mutant astrocytoma and oligodendroglioma patients, WHO grade 2 and 3, undergoing resection (*n* = 50) or non-surgical treatment (*n* = 98) at 1^st^ recurrence between 2001 and 2023. In surgical cases, FLAIR tumor volumes (TV) were assessed volumetrically. Prognostic factors were identified by log-rank tests and multivariable Cox proportional hazards regression. Median follow-up was 140 months.

**Results:**

Female sex (*p* = 0.005), frontal tumors (*p* = 0.029) and iterative resections (*p* = 0.025) were more frequent in surgical patients, while non-surgical patients received more systemic treatment. Functional status (KPS, NANO) and tumor characteristics (histology, WHO grade) were balanced. In multivariable analysis, re-resection was associated with prolonged PFS-2 (*p* = 0.029, HR = 0.560, 95% CI 0.332–0.944), but not with SAR. In surgical cases, median pre- and postoperative TVs were 19.75 cm^3^ and 4.545 cm^3^ (*p* < 0.00001). PFS-2 was significantly prolonged in patients without (*n* = 12) compared to surgical patients with residual TV (*n* = 38; *p* = 0.022) and all patients with residual disease, including non-surgical cases (*n* = 136; *p* = 0.007). Following re-resection, a permanent deficit remained in 1 patient (2%). Functional status was preserved, with stable rates of KPS ≥90% and NANO = 0 at 7 days and 3 months postoperatively.

**Conclusion:**

In this cohort of IDH-mutant lower-grade gliomas, repeat resection at 1^st^ recurrence was safe and associated with prolonged PFS-2, especially when GTR was achieved, supporting its relevance in multimodal treatment.

**Supplementary Information:**

The online version contains supplementary material available at 10.1007/s11060-025-05353-x.

## Introduction

Diffuse astrocytoma, isocitrate dehydrogenase mutant (IDHmut), and oligodendroglioma, IDHmut, 1p/19q co-deleted, confer prognostically favorable glioma subtypes [1]. According to the World Health Organization (WHO) classification, neuropathological diagnosis is based on combined histological and molecular information, with a panel of subtype-defining molecular markers required for a diagnosis consistent with WHO CNS 5 [2]. These “lower-grade gliomas” comprise both WHO grade 2 and 3 tumors. While the grading has historically influenced adjuvant treatment [3], its prognostic impact and diagnostic feasibility has been challenged lately [4–7]. At 1^st^ diagnosis, early surgical resection is a cornerstone of treatment. For IDHmut glioma WHO grade 2, long-term tumor control was observed when maximal safe resection was achieved [8–10], with a subtype-dependent impact of the extent of resection (EOR) still discussed [10]. At recurrence, treatment is not standardized and should be tailored to the patient’s clinical condition, tumor characteristics and pre-treatments [3]. Repeat resection offers the opportunity to alleviate neurological symptoms and seizures, assess malignant transformation and guide molecularly-informed treatment [11]. However, a potential survival benefit is still debated since previous studies lacked comprehensive molecular characterization or quantitative assessment of residual tumor volume (RTV) [12–16]. Because lower-grade glioma patients experience long-term survival, extended follow-up introduces additional bias as treatment paradigms evolve. Recently updated evidence-based guidelines by the Joint Tumor Section of the American Association of Neurological Surgeons (AANS) and the Congress of Neurological Surgeons (CNS) addressed the role of surgery for recurrent glioma WHO grade 2 [17]. Only two retrospective studies with class III evidence qualified for reporting [14, 15], but data were not sufficient to recommend surgery for improving outcome. Hence, we investigated whether resection at 1^st^ recurrence – especially gross total resection (GTR) - compared to non-surgical treatment is associated with progression-free survival after recurrence (PFS-2) and survival after recurrence (SAR) in a molecularly characterized cohort of recurrent lower-grade glioma patients with volumetric quantification of the EOR. We also evaluated surgical complications and functional outcomes associated with repeat resection.

## Methods

### Patient sample

Consecutive patients with diffuse glioma WHO grade 2 and 3, treated at the Department of Neurosurgery, University Hospital Heidelberg, between 1992 and 2023 were identified from surgical records, screened for tumor recurrence and stratified by whether undergoing re-resection (“surgical cohort”) or radiotherapy and/or systemic treatment (“non-surgical cohort”) at 1^st^ recurrence (Supplementary Figure S1). Confirmed IDH mutation (immunohistochemistry/sequencing) and availability of subtype-defining molecular markers were a prerequisite. In all but 11 patients, diagnosis was reported or re-classified according to WHO CNS 5 [2]. In the latter, diagnosis was made according to WHO 2016 [18]. Because of the anticipated poor prognosis [19], astrocytoma patients with cyclin-dependent kinase inhibitor (CDKN) 2A/B homozygous deletion were excluded. Patients were also excluded when lost to follow-up, refused treatment, treated elsewhere or when re-resection revealed no vital tumor. For the surgical cohort, digital availability of magnetic resonance (MR) imaging was required. Pre- and early postoperative (≤48 hours; epMRI) MRI was performed at 3Tesla using a standardized protocol including fluid-attenuated inversion recovery (FLAIR)-weighed (w), T2-w and T1-w sequences before and after application of contrast agent. Demographic and clinical data (new/aggravated neurological deficits or seizures (“symptomatic recurrence”); Karnofsky Performance Scale (KPS) and Neurologic Assessment in Neuro-Oncology (NANO) scores; treatment modalities) at 1^st^ recurrence were collected retrospectively. For illustration of treatment heterogeneity, proportions of patients with treatment start at 1^st^ recurrence across different study eras were compared: (1) 2001–2010 (pre-IDH era); 2011–2016 (landmark trials influencing treatment standards [20–22]); (3) 2017–2021 (following WHO 2016); (4) 2022–2023 (following WHO CNS 5). Follow-up was through March 11th, 2025.

### Volumetric analysis

For the surgical cohort, semi-automated segmentation of tumor volumes (TV) using the Brainlab™ software SmartBrush version 4.5 (Brainlab, Germany) was performed by two experienced raters (KG, CJ) on FLAIR-w MR. EOR was reported in % and RTV in cm^3^, with GTR defined as no RTV. All GTR cases were independently confirmed by both raters.

### Statistical analysis

Statistical analysis was conducted using SPSS Statistics (version 29.0.0.0, IBM Corp., Armonk, USA) and GraphPad Prism (version 10.5.0, GraphPad Software, Inc., La Jolla, USA). Categorial and continuous variables were compared by Fisher’s exact and Chi-square tests or two-sided Mann-Whitney tests, respectively. Follow-up was calculated using the reverse Kaplan-Meier method. Survival analysis was performed using univariate log-rank tests and a multivariable Cox proportional hazards (CPH) model with stepwise forward selection of confounding variables that were included based on univariate significance or clinical relevance (sex; age at recurrence; symptomatic recurrence; KPS, NANO, lateralization, localization, eloquence, histology and WHO grade at recurrence; repeat resection yes/no). To account for potential lead-time bias, time from recurrence imaging until treatment start was also included. Only cases without missing data were analyzed. Overall survival (OS) was defined from the time of 1^st^ surgery until death or censored to last follow-up, PFS-2 from radiographic recurrence until second progression or censored to death or last follow-up, and SAR from radiographic recurrence until death or censored to last follow-up. Given the long observation period, a uniform definition of radiographic recurrence was not feasible. Most cases were evaluated using RANO criteria, supplemented by tumor board consensus. The significance level was set at 0.05.

## Results

### Patient characteristics

**Fig. 1:**
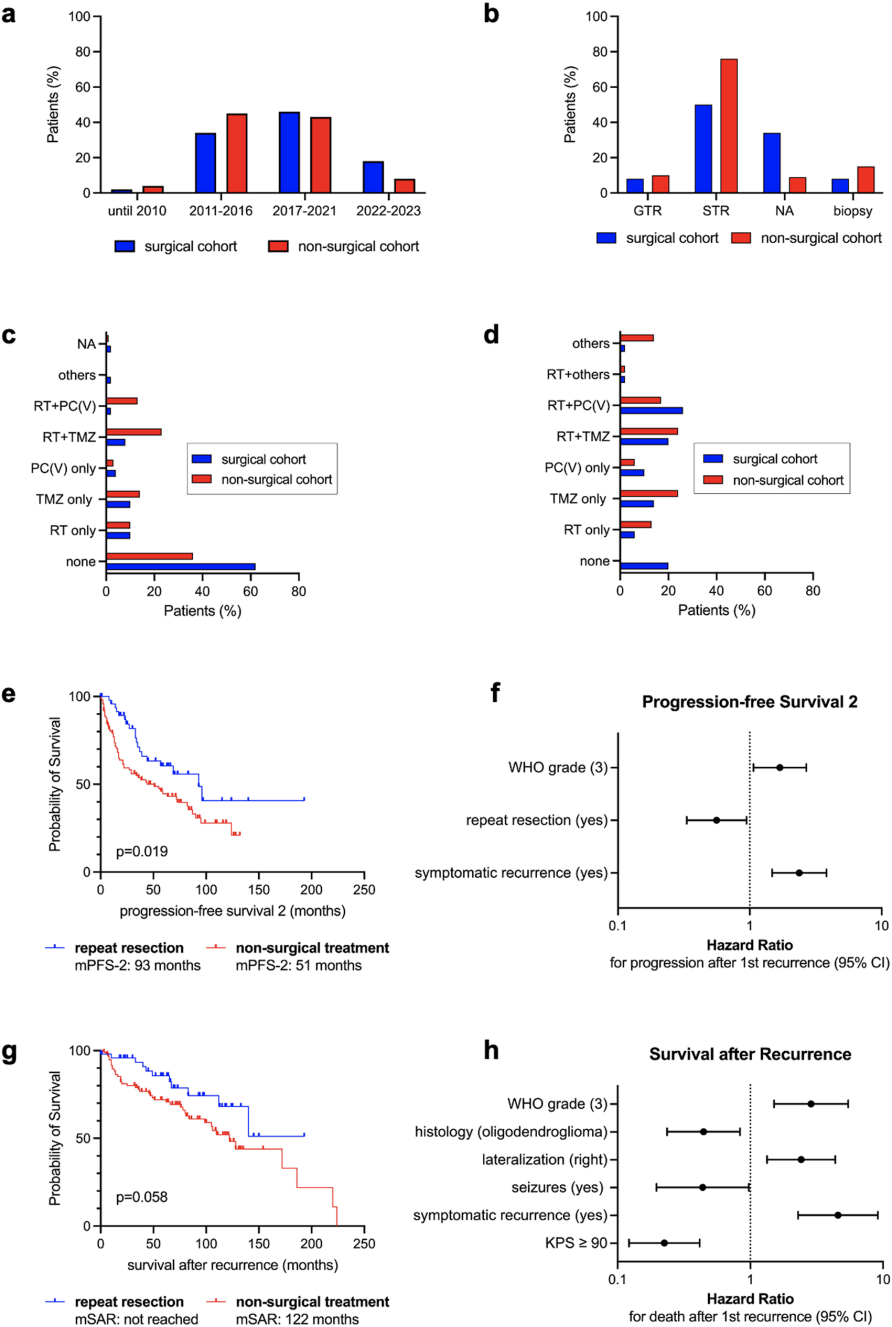
Treatment characteristics and prognostic relevance of repeat resection at 1st recurrence compared to non-surgical treatment in IDHmut lower-grade glioma patients (n=148). (a-d) Treatment characteristics stratified by treatment groups at 1st recurrence (surgical vs. non-surgical cohort). (a) Comparable proportions of patients starting treatment at 1st recurrence across different study eras (p=0.246; Chi-square test). (b) Extent of resection categories at 1st diagnosis differed significantly between treatment groups (p<0.001 for comparing GTR vs. subtotal resection (STR) vs. EOR not available (NA); Chi-square test). (c,d) Illustration of adjuvant treatment at 1st diagnosis (c) and at 1st recurrence (d). RT: radiotherapy; TMZ: temozolomide; PC(V): procarbazine, CCNU, (vincristine) (e,g). Kaplan-Meier plots depicting progression-free survival 2 (PFS-2; e) and survival after recurrence (SAR; g) stratified by treatment at 1st recurrence with 50 patients undergoing repeat resection and 98 patients receiving non-surgical treatment. Note that median (m) PFS-2 was significantly prolonged in the surgical cohort. (f,h) Forest plots displaying results from multivariable survival analysis employing Cox proportional hazards regression with clinical variables included based on univariate statistical significance or clinical relevance. Only prognostic factors reaching statistical significance (p≤0.05) are presented for PFS-2 (f) and SAR (h). Hazard ratios of death (SAR) or 2nd progression (PFS-2) along with 95% confidence interval (CI) are displayed on the x-axis. Note that the x-axis is log transformed. Reference levels are given in brackets. For detailed information on univariate log-rank and multivariable Cox regression p-values, see Table 2 and Suppl. Table 1.

148 patients (83 males, 65 females) with radiographically confirmed 1^st^ recurrence of IDHmut astrocytoma (*n* = 66; 44.6%) or oligodendroglioma (*n* = 82; 55.4%) were analyzed (Table 1). WHO grade 2 (49.3%) and 3 (50%) tumors were balanced. At 1^st^ diagnosis, most patients (*n* = 130; 87.8%) underwent resection as opposed to biopsy (*n* = 18; 12.2%) and 54.1% (*n* = 80) received adjuvant treatment. At 1^st^ recurrence, median age was 42 years (range 22–82). KPS prior to treatment was high (median 90; range 50–100) and most patients (*n* = 103; 69.6%) were neurologically intact (NANO score 0). 50 patients (33.8%) underwent repeat resection, followed by adjuvant treatment in 40 cases (80%), while 98 patients (66.2%) underwent non-surgical treatment. Treatment decisions were based on multidisciplinary consensus (tumor board recommendations in 33.1%). Treatment start at 1^st^ recurrence was between 2001 and 2023, with comparable proportions of patients treated across different study eras (*p* = 0.246; Table 1; Fig. 1a). Median time from radiographic recurrence until treatment was 31 days (range 1–312) in surgical and 28 days (range 1–954) in non-surgical patients (*p* = 0.436; Table 1). Treatment groups were comparable regarding histology (*p* = 0.163) and WHO grade (*p* = 0.385). CDKN2A/B status was available in 63.5%. Following repeat resection, malignant transformation to WHO grade 3 was observed in 5 patients (10%). Female sex (60% vs. 35.7%; *p* = 0.005), frontal tumors (86% vs. 69.4%; *p* = 0.029) and iterative resections (86% vs. 69.4%; *p* = 0.029) were more frequent in the surgical cohort. In contrast, a trend towards larger RTV at 1^st^ diagnosis was observed in the non-surgical cohort (2.33 vs. 7.28 cm^3^; *p* = 0.054; Table 1) and the distribution of EOR categories differed significantly between groups (*p* < 0.0001; Table 1; Fig. 1b). Also, more patients of the non-surgical cohort received adjuvant treatment at 1^st^ diagnosis (63.3% vs. 36%; *p* = 0.003) or any kind of systemic treatment after 1^st^ recurrence (86.7% vs. 72%; *p* = 0.042) and any further progression (*p* = 0.007) (Table 1; Fig. 1c–d). Importantly, variables suggesting a potential selection bias (lateralization (*p* = 0.292), eloquence (30% vs. 36.7%; *p* = 0.468), symptomatic recurrence (28% vs. 26.5%; *p* = 1.0), KPS and NANO scores prior to treatment) were comparable between groups (Table 1).

**Table 1:** Patient characteristics

Variable	All Patients (n=148)	Re-Resection (n=50)	No Re-Resection (n=98)	p-value
Sex (n; %)				0.005*
- male	83 (56.1)	20 (40)	63 (64.3)	
- female	65 (43.9)	30 (60)	35 (35.7)	
**At 1**^**st**^ **diagnosis**				
Type of surgery				0.303*
- Resection	130 (87.8)	46 (92)	84 (85.7)	
- Biopsy	18 (12.2)	4 (8)	14 (14.3)	
Extent of resection^%^				
- GTR (RTV 0 cm^3^)	5 (3.8)	4 (8.7)	1 (1.2)	**<0.0001°**
- STR (RTV >0 cm^3^)	99 (76.2)	25 (54.3)	74 (88.1)	
- RTV not available	26 (20)	17 (37)	9 (10.7)	
Volume FLAIR post-op^%^(cm3; median (range))	5.77 (0-180.1)	2.33 (0-180.1)	7.78 (0-102.5)	0.054^&^
Adjuvant treatment				0.003*
- yes	80 (54.1)	18 (36)	62 (63.3)	
- no	66 (44.6)	31 (62)	35 (35.7)	
- NA	2 (1.3)	1 (2)	1 (1)	
Type of adjuvant treatment				NA
- Radiotherapy (RT) only	15 (10.1)	5 (10)	10 (10.2)	
- TMZ only	19 (12.8)	5 (10)	14 (14.3)	
- PC(V) only	5 (3.4)	2 (4)	3 (3.1)	
- RT + TMZ	26 (17.6)	4 (8)	22 (22.4)	
- RT + PC(V)	14 (9.5)	1 (2)	13 (13.3)	
- others	1 (0.7)	1 (2)	0 (0)	
- none	66 (44.6)	31 (62)	35 (35.7)	
- NA	2 (1.3)	1 (2)	1 (1)	
**At 1**^**st**^ **recurrence**				
Interval from recurrence imaging to treatment start (days; median (range))	28 (1-954)	31 (1-312)	28 (1-954)	0.436^&^
Treatment decisions based on tumor board (n; %)				0.712°
- yes	49 (33.1)	18 (36)	31 (31.6)	
- no	99 (66.9)	32 (64)	67 (68.4)	
Eras by treatment start at 1^st^ recurrence (n; %)				0.246°
- 2001 - 2010	5 (3.4)	1 (2)	4 (4.1)	
- 2011 - 2016	61 (41.2)	17 (34)	44 (44.9)	
- 2017 - 2021	65 (43.9)	23 (46)	42 (42.9)	
- 2022 - 2023	17 (11.5)	9 (18)	8 (8.1)	
Age (years; median (range))	42 (22-82)	43 (25-79)	41 (22-82)	0.56^&^
Histology				0.163*
- astrocytoma, IDH mutant	66 (44.6)	18 (36)	48 (49)	
- oligodendroglioma	82 (55.4)	32 (64)	50 (51)	
WHO grade				0.385*
- WHO grade 2	73 (49.3)	27 (54)	46 (46.9)	
- WHO grade 3	74 (50)	22 (44)	52 (53.1)	
- NA	1 (0.7)	1 (2)	0 (0)	
Malignant transformation	NA		NA	NA
- yes		5 (10)		
- no		45 (90)		
CDKN2A/B				NA
- intact	71 (48)	20 (40)	51 (52)	
- heterozygous deletion	17 (11.5)	6 (12)	11 (11.2)	
- homozygous deletion^1^ (oligodendroglioma only)	5 (3.4)	1 (2)	4 (4.1)	
- gain	1 (0.7)	1 (2)	0 (0)	
- NA	54 (36.5)	22 (44)	32 (32.7)	
(NA: astrocytoma)	25 (46.3%)	8 (36.4%)	17 (53.1)	
(NA: oligodendroglioma)	29 (53.7%)	14 (63.6%)	15 (46.9)	
Tumor lateralization				0.292°
- right	72 (48.6)	26 (52)	46 (46.9)	
- left	75 (50.7)	23 (46)	52 (53.1)	
- bilateral	1 (0.7)	1 (2)	0 (0)	
Tumor localization				0.029*
- frontal	111 (75)	43 (86)	68 (69.4)	
- others	37 (25)	7 (14)	30	
Tumor eloquence				0.468*
- yes	51 (34.5)	15 (30)	36 (36.7)	
- no	97 (65.5)	35 (70)	62 (63.3)	
Contrast enhancement				0.116*
- yes	73 (49.3)	30 (60)	43 (43.9)	
- no	72 (48.7)	20 (40)	52 (53.1)	
- NA	3 (2)	0 (0)	3 (3)	
KPS (median (range))	90 (50-100)	90 (70-100)	90 (50-100)	0.131^&^
KPS ≥ 90				0.393*
- yes	116 (78.4)	42 (84)	74 (75.5)	
- no	31 (20.9)	8 (16)	23 (23.5)	
- NA	1 (0.7)	0	1 (1)	
NANO = 0				0.342
- yes	103 (69.6)	38 (76)	65 (66.3)	
- no	44 (29.7)	12 (24)	32 (32.7)	
- NA	1 (0.7)	0 (0)	1 (1)	
Symptomatic recurrence				1.0*
- yes	40 (27)	14 (28)	26 (26.5)	
- no	107 (72.3)	36 (72)	71 (72.5)	
- NA	1 (0.7)	0 (0)	1 (1)	
Seizures				0.814*
- yes	24 (16.2)	9 (18)	15 (15.3)	
- no	123 (83.1)	41 (82)	82 (83.7)	
- NA	1 (0.7)	0 (0)	1 (1)	
Resection	50 (33.8)	50 (100)	0 (0)	NA
- with adjuvant treatment		40 (80)		
- w/o adjuvant treatment		10 (20)		
Radiotherapy				0.123*
- yes	41 (27.7)	18 (36)	23 (23.5)	
- no	107 (72.3)	32 (64)	75 (76.5)	
Systemic therapies				0.042*
- yes	121 (81.8)	36 (72)	85 (86.7)	
- no	27 (18.2)	14 (28)	13 (13.3)	
**After 2**^**nd**^ **progression**				
Further recurrences (n; median (range))	1 (0-6)	0 (0-6)	1 (0-6)	**0.012** ^**&**^
Further resections (n; median (range))	0 (0-3)	0 (0-3)	0 (0-2)	**0.025** ^**&**^
Further radiation therapies (n; median (range))	0 (0-3)	0 (0-2)	0 (0-3)	0.641^&^
Further systemic therapies (n; median (range))	0 (0-6)	0 (0-3)	0 (0-6)	**0.007** ^**&**^
**Outcome Parameters**				
Deaths (n; %)	54 (36.5)	11 (22)	43 (43.9)	0.011*
2^nd^ progression (n; %)	78 (52.7)	19 (38)	59 (60.2)	0.015*
Overall survival (months; median (range))	219 (9-362)	300 (32-362)	186 (9-318)	**<0.001** ^**§**^
Progression-free survival 2 (months; median (range))	63 (0-193)	93 (0-193)	51 (0-132)	**0.019** ^**§**^
Survival after recurrence (months; median (range))	140 (0-224)	nr (0-193)	122 (3-224)	0.058^§^
Follow-up (months; median (range))	140 (9-362)	148 (32-362)	136 (9-318)	NA

Patients with recurrent IDHmut lower-grade glioma are presented as the complete cohort (*n* = 148) and by treatment groups at 1st tumor recurrence. p-values relate to the comparison of patients with (*n* = 50) and without (*n* = 98) repeat resection and are given in bold if significance level was reached (≤0.05). NA = not available; NR = not reached; ^1^homozygous deletion relates to oligodendroglioma patients only; * Fisher’s exact test; ° Chi-square test; ^&^ Mann-Whitney test; ^§^ Log-rank test; ^%^ patients with resection at 1^st^ diagnosis only (*n* = 130)

**Table 2:** Multivariable analysis of progression-free survival 2 and survival after recurrence in recurrent IDHmut lower-grade glioma patients

Variables	p-value	HR	95% CI
**Progression-free survival 2** (in days; *n* = 140)			
Symptomatic recurrence (yes)	< 0.001	2.374	1.476 - 3.818
Repeat resection (yes)	0.029	0.560	0.332 - 0.944
WHO grade rec (WHO 3)	0.025	1.693	1.068 - 2.683
**Survival after recurrence** (in days; *n* = 141)			
KPS rec ≥90 (yes)	< 0.001	0.225	0.122 - 0.415
Symptomatic recurrence (yes)	< 0.001	4.572	2.289 - 9.132
Histology rec (oligodendroglioma)	0.012	0.444	0.236 - 0.836
WHO grade rec (WHO 3)	0.001	2.869	1.506 - 5.463
Seizures rec (yes)	0.043	0.437	0.196 - 0.974
Lateralization rec (right vs. left)	0.004	2.414	1.336 - 4.363

Factors associated with progression-free survival 2 (PFS-2) and survival after recurrence (SAR) were identified by multivariable Cox proportional hazards regression. The following categorial (cat.) and continuous (cont.) variables were included in the model based on univariate significance and clinical relevance: sex (cat.); age rec (cont.); KPS rec ≥90 (cat.); NANO rec = 0 (cat.); symptomatic recurrence (cat.); seizures (cat.); tumor lateralization rec (right vs. left; cat.); localization rec (frontal vs. others; cat.); eloquence rec (cat.); repeat resection (cat.); histology rec (cat.); WHO grade rec (cat.); time from recurrence imaging until treatment start (cont.). Reference levels are given in brackets. HR: hazard ratio; CI: confidence interval; rec: at recurrence

### Prognostic relevance of repeat resection compared to non-surgical treatment

Over a median follow-up of 140 months (range 9–362), 54 patients (36.5%) deceased, and 78 patients (52.7%) experienced subsequent progression. For all patients, median (m) OS was 219 months (range 9–362), PFS-2 was 63 months (range 0–193) und SAR was 140 months (range 0–224) (Table 1).

In univariate analysis, repeat resection was associated with prolonged mPFS-2 (93 vs. 51 months; *p* = 0.019) and showed a trend towards prolonged mSAR (not reached vs. 122 months; *p* = 0.058) (Fig. 1e, g). Median OS also differed between groups (300 vs. 186 months; *p* < 0.001; Supplementary Figure S2a) but was not further investigated because of immortal-time bias.

Other factors predictive of PFS-2 and SAR are listed in Suppl. Table S1 and were included in a multivariable CPH model. Herein, repeat resection was associated with prolonged PFS-2 (*p* = 0.029; HR = 0.560; 95% CI 0.332–0.944), but not SAR. Other predictors were: KPS ≥ 90 (SAR: *p* < 0.001, HR = 0.225), symptomatic recurrence (PFS-2: *p* < 0.001, HR = 2.374; SAR: *p* < 0.001, HR = 4.572), seizures (SAR: *p* = 0.043, HR = 0.437), lateralization (SAR: *p* = 0.004, HR = 2.414), histology (SAR: *p* = 0.012, HR = 0.444) and WHO grade (PFS-2: *p* = 0.025, HR = 1.693; SAR: *p* = 0.001, HR = 2.869) (Table 2; Fig. 1f, h).

Notably, 18 patients of the non-surgical cohort underwent re-resection at another recurrence. There was a trend towards prolonged mSAR in patients with “ever repeat resection” (*n* = 68) compared to patients with “never repeat resection” (*n* = 80) (172 vs. 122 months; *p* = 0.09; Supplementary Figure S2b).

### Residual tumor volumes associated with improved outcome

**Fig. 2:**
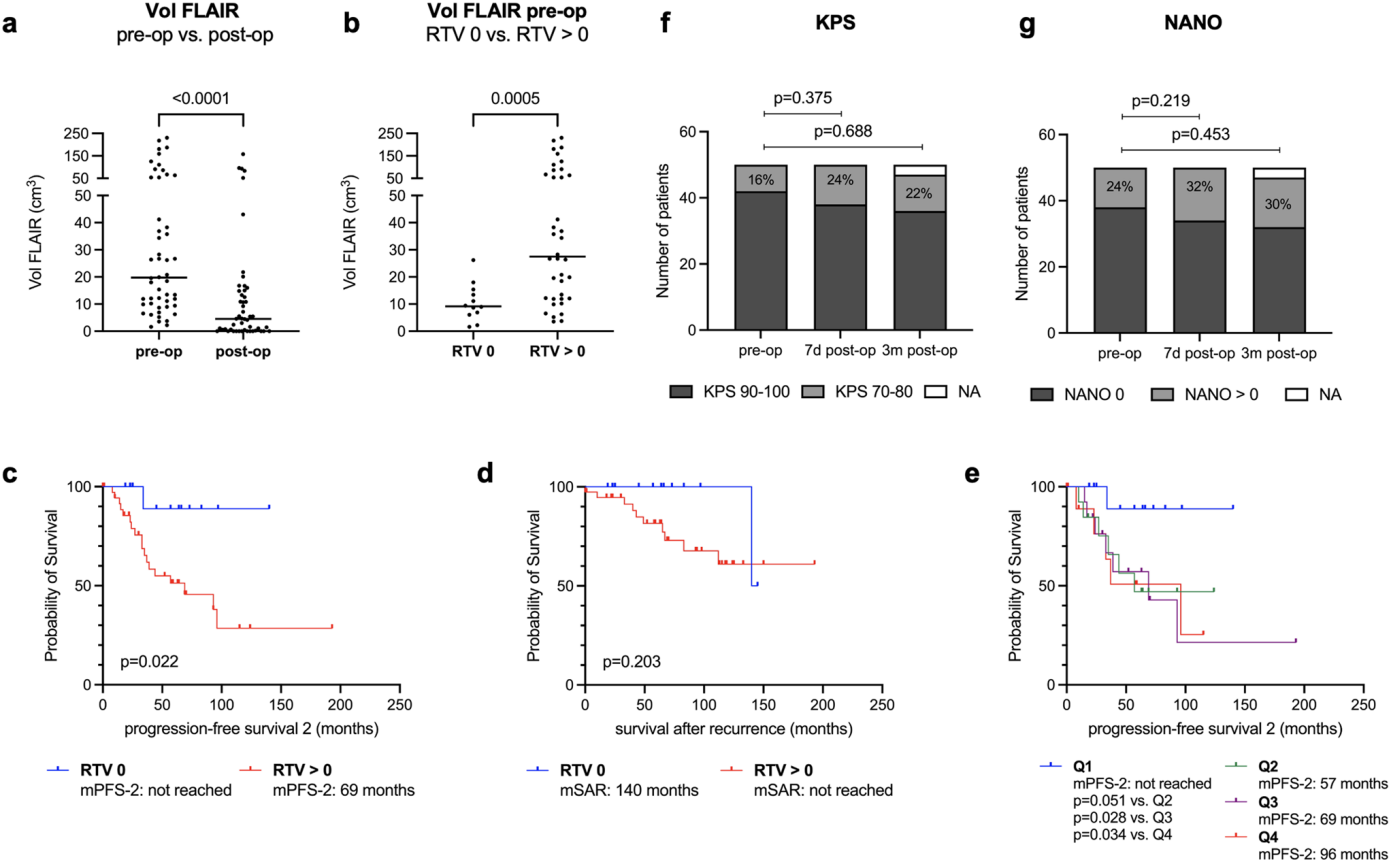
Tumor volumetry, survival and functional outcomes in IDHmut lower-grade glioma patients undergoing repeat resection at 1st recurrence (n=50). (a) FLAIR tumor volumes (Vol FLAIR) displayed by scatter dot plot (with every dot representing a single patient) were significantly reduced from preoperative (pre-op; median 19.75 cm3, range 1.59-230.4 cm3) to early postoperative (post-op; median 4.545 cm3, range 0-157.4 cm3) MRI. (b) Pre-op Vol FLAIR was significantly smaller in patients with gross total resection (n=12; residual tumor volume (RTV)=0 cm3) compared to patients with any RTV (median 9.195 cm3 vs. 27.5 cm3). (c-d) Kaplan-Meier plots depicting progression-free survival 2 (PFS-2; c) and survival after recurrence (SAR; d) stratified by RTV. Patients with no RTV conferred significantly prolonged median (m) PFS-2 and SAR. (e) Stratifying patients by postoperative FLAIR tumor volume quartiles (Q), Q1 (0 cm3) was associated with prolonged mPFS-2 compared to all other quartiles (Q2: 0.21-4.49 cm3; Q3: 4.6-14.8 cm3; Q4: 15.0-157.4 cm3). Besides the clear survival benefit associated with no RTV, no further resection threshold could be established. (f-g) Functional outcomes of repeat resections are illustrated by Karnofsky Performance Score (KPS; f) and Neurologic Assessment in Neurooncology (NANO; g) score over time (pre-op, 7 days (d) post-op and 3 months (m) post-op). Patient numbers are displayed on the y-axis with selected proportions of patients (%) given within the respective bars. Note that the proportion of patients with KPS≥90 and NANO=0 (no neurological deficits) remained unchanged over time. P-values are given for paired Wilcoxon tests.

Since PFS-2 may correlate with the EOR, we performed volumetric analysis for the surgical cohort (*n* = 50). Median preoperative FLAIR TV was 19.75 cm^3^ (range 1.59–230.4 cm^3^) which was reduced to a median RTV of 4.545 cm^3^ (range 0–157.4 cm^3^) on epMRI (Fig. 2a). Notably, in some cases with very large tumors, tumor debulking rather than maximal resection was intended. Accordingly, the EOR ranged from 20.5% to 100% (median 85.2%). Nevertheless, GTR was achieved in 12 patients (24%). Compared to patients with any RTV (*n* = 38; 76%), median preoperative TVs were significantly smaller (9.195 cm^3^ vs. 27.5 cm^3^; *p* = 0.0005; Fig. 2b) and the proportion of tumors with eloquent location (0% vs. 39.5%; *p* = 0.01) and contrast enhancement (25% vs. 71.1%; *p* = 0.007) was significantly lower. Histology (*p* = 0.089) and WHO grade (*p* = 0.303) were comparable. Also, similar proportions of patients underwent adjuvant treatment (83.3% vs. 78.9%; *p* = 1.0) (Table 3). GTR significantly prolonged mPFS-2 (not reached vs. 69 months; *p* = 0.022), but not mSAR (140 months vs. not reached; *p* = 0.203) (Fig. 2c, d). When comparing patients after GTR to all patients with residual tumor, including those with non-surgical treatment, mPFS-2 was significantly prolonged as well (not reached vs. 55 months; *p* = 0.007), while mSAR showed a trend towards improved outcome (140 vs. 128 months; *p* = 0.099) (Suppl. Figure S2c-S2d).

**Table 3:** Patient characteristics, surgical details and volumetric analysis of the surgical cohort

Variable	All patients (n=50)	RTV 0 cm^3^ (n=12)	RTV > 0 cm^3^ (n=38)	p-value
Sex (n; %)				0.317*
- male	20 (40)	3 (25)	17 (44.7)	
- female	30 (60)	9 (75)	21 (55.3)	
**At 1** ^**st**^ ** recurrence**				
Age (years; median (range))	43 (25-79)	41.5 (25-63)	43 (25-79)	0.594^&^
Histology				0.089*
- astrocytoma, IDH mutant	18 (36)	7 (36)	11 (28.9)	
- oligodendroglioma	32 (64)	5 (64)	27 (71.1)	
WHO grade				0.303*
- WHO grade 2	27 (54)	8 (66.7)	19 (50)	
- WHO grade 3	22 (44)	3 (25)	19 (50)	
- NA	1 (2)	1 (8.3)	0 (0)	
Malignant transformation				0.582*
- yes	5 (10)	2 (16.7)	3 (7.9)	
- no	45 (90)	10 (83.3)	35 (92.1)	
Tumor lateralization				0.501°
- right	26 (52)	5 (52)	21 (46.9)	
- left	23 (46)	7 (46)	16 (53.1)	
- bilateral	1 (2)	0 (2)	1 (0)	
Tumor localization				1.0*
- frontal	43 (86)	10 (83.3)	33 (86.8)	
- others	7 (14)	2 (16.7)	5 (13.2)	
Tumor eloquence				0.01*
- yes	15 (30)	0 (0)	15 (39.5)	
- no	30 (70)	12 (100)	23 (60.5)	
Contrast enhancement				0.007*
- yes	30 (60)	3 (25)	27 (71.1)	
- no	20 (40)	9 (75)	11 (28.9)	
Volume FLAIR pre-op (cm^3^; median (range))	19.75 (1.59-230.4)	9.195 (1.59-26.2)	27.5 (3.57-230.4)	**0.0005** ^**&**^
Volume FLAIR post-op (cm^3^; median (range))	4.545 (0-157.4)	0	8.19 (0.21-157.4)	NA
Volume FLAIR post-op (cm^3^; quartiles)		NA	NA	NA
- Q1 (0-25%)	0			
- Q2 (25-50%)	0.21-4.49			
- Q3 (50-75%)	4.6-14.8			
- Q4 (75-100%)	15.0-157.4			
EOR (%; median (range))	85.2 (20.5-100)	100	79.7 (20.5-96.6)	**<0.0001** ^**%**^
Symptomatic recurrence				0.14*
- yes	14 (28)	1 (8.3)	13 (34.2)	
- no	36 (72)	11 (91.7)	25 (65.8)	
Neurological deterioration				0.48*
- no	44 (88)	12 (100)	32 (84.2)	
- yes, resolved within 30 days	5 (10)	0 (0)	5 (13.2)	
- yes, persistent	1 (2)	0 (0)	1 (2.6)	
Revision surgery				0.426*
- yes	2 (4)	1 (8.3)	1 (2.6)	
- no	48 (96)	11 (91.7)	37 (97.4)	
iMRI				1.0*
- yes	47 (94)	12 (100)	35 (92.1)	
- no	3 (6)	0 (0)	3 (7.9)	
IOM				0.621*
- yes	6 (12)	2 (16.7)	4 (10.5)	
- no	44 (88)	10 (83.3)	34 (89.5)	
Awake surgery				0.379*
- yes	8 (16)	3 (25)	5 (13.2)	
- no	42 (84)	9 (75)	33 (86.8)	
Resection				1.0*
- with adjuvant treatment	40 (80)	10 (83.3)	30 (78.9)	
- w/o adjuvant treatment	10 (20)	2 (16.7)	8 (21.1)	
**Outcome Parameters**				
Deaths (n; %)	11 (22)	1 (8.3)	10 (26.3)	0.257*
2^nd^ progression (n; %)	19 (38)	1 (8.3)	18 (36)	0.018*
Overall survival (months; median (range))	300 (32-362)	252 (64-271)	300 (32-362)	0.259^§^
Progression-free survival 2 (months; median (range))	93 (0-193)	nr (19-140)	69 (0-193)	**0.022** ^**§**^
Survival after recurrence (months; median (range))	nr (0-193)	140 (19-145)	nr (0-193)	0.203^§^

Patients are presented based on the amount of FLAIR-hyperintense residual tumor volume (RTV), with p-values given in bold if significance level was reached (≤0.05). Q = quartile; NR = not reached; * Fisher’s exact test; ° Chi-square test; ^&^ Mann-Whitney test; ^%^ Wilcoxon signed rank test; ^§^ log-rank test

Due to the large range of TVs, a reliable resection threshold with prognostic impact could not be determined. When RTV was stratified by quartiles (Q), no RTV (Q1) was associated with prolonged PFS-2 compared to all other quartiles (Q2: 0.21–4.49 cm^3^; Q3: 4.6–14.8 cm^3^; Q4: 15.0–157.4 cm^3^). However, no significant PFS-2 difference was observed when Q2-4 were compared against each other (Fig. 2e).

When RTV was further stratified by tumor subtypes, mPFS-2 was longest in oligodendroglioma and astrocytoma patients without RTV (not reached), followed by oligodendroglioma and astrocytoma patients without RTV (93 and 57 months, respectively) (Suppl. Figure S2e).

### Risk profile of repeat resections

For maximal safe resection, intraoperative MRI was used in 94% (*n* = 47), awake surgery in 16% (*n* = 8) and asleep surgery guided by intraoperative neuromonitoring (IONM) in 12% (*n* = 6) (Table 3). After surgery, 6 patients (12%), all with RTV, experienced neurological deterioration, presenting with a new motor deficit in all cases, but only 1 patient remained with a permanent motor deficit (2%) (Table 3). Accordingly, the proportions of patients with KPS ≥ 90 and NANO = 0 remained stable comparing the preoperative situation to the status at 7 days (KPS: *n* = 42 vs. 38, *p* = 0.375; NANO: *n* = 38 vs. 34, *p* = 0.688) and 3 months (KPS: *n* = 42 vs. 36, *p* = 0.219; NANO: *n* = 38 vs. 32, *p* = 0.453) postoperatively (Fig. 2f,g). Surgical revision for a postoperative complication was required in 2 patients (4%): one due to cerebrospinal fluid (CSF) fistula and one due to impaired wound healing (Table 3). In two additional patients (4%), subcutaneous CSF collections resolved spontaneously.

## Discussion

In this cohort of molecularly characterized recurrent lower-grade glioma, repeat resection at 1^st^ recurrence was associated with prolonged PFS-2 (median 7.75 years) and low rates of neurological (2%) and surgical (4%) complications. Particularly, GTR prolonged PFS-2 compared to any incomplete resection or non-surgical treatment.

At 1^st^ diagnosis, the prognostic benefit of maximized resections is established, although interpretation must consider evolving diagnostic criteria for lower-grade gliomas. Recently, Hervey-Jumper et al. analyzed the combined effects of TVs, molecular factors and adjuvant treatment on outcome in IDHmut astrocytoma and oligodendroglioma WHO grade 2 [8]. Across both subtypes, EOR ≥75% improved OS, while EOR ≥80% improved PFS.

At recurrence, the evidence supporting reoperation is sparse. The AANS/CNS guidelines for recurrent glioma WHO grade 2 concluded that data were insufficient to recommend repeat resection for improving outcome [17]. Ramakrishna et al. reported on 52 WHO grade 2 glioma patients undergoing reoperation [14]. Median OS (12.95 years) was prolonged in patients without residual FLAIR tumor after 1^st^ or 2^nd^ resection. Malignant transformation to WHO grade 3 or 4 (22 patients) and residual tumor after reoperation were associated with shorter PFS-2. Spitaels et al. reported on 35 WHO grade 2 glioma patients, of whom 25 underwent repeat resection [15]. Malignant transformation was noted in 6 cases. PFS-2 was significantly prolonged in patients with adjuvant treatment after repeat resection or non-surgical treatment compared to watchful waiting. However, volumetric EOR and molecular status including IDH were not investigated in both studies. Shofty et al. observed prolonged OS (11.2 vs. 5.5 years) and delayed malignant transformation in reoperated IDHmut patients (*n* = 50) compared to non-surgical patients (*n* = 22) [16].

In our cohort, GTR at reoperation was significantly associated with prolonged PFS-2, consistent with Ramakrishna et al. [14], compared to any incomplete resection or non-surgical treatment. Notably, patients with GTR had smaller preoperative FLAIR TVs in non-eloquent areas, making them ideal candidates for maximal safe resections. In newly diagnosed IDHmut glioma, preoperative TVs correlate with resectability and survival [8, 23]. Notably, our study included patients independent of surgical intent, yielding a wide range of preoperative TVs (1.59–230.4 cm^3^), RTVs (0 to 157.4 cm^3^) and EOR (20.46% to 100%). Hence, a prognostic resection threshold could not be defined, beyond the observed survival benefit of GTR. Interestingly, GTR was associated with improved PFS-2 in both tumor subtypes, consistent with observations in newly diagnosed IDHmut gliomas [10]. Larger cohorts with uniform resection goals are needed to clarify whether a (subtype-dependent) resection threshold exists.

Irrespective of EOR, re-resection at 1^st^ recurrence was associated with prolonged PFS-2 (HR = 0.560; *p* = 0.029) in multivariable-adjusted analysis and showed a trend towards improved SAR. PFS-2 was almost doubled compared to non-surgical treatment (93 vs. 51 months; *p* = 0.019). Despite including WHO grade 3 tumors, outcomes of our surgical cohort (median OS 25 years, PFS-2 7.75 years) compare favorably to other studies, likely due to strict inclusion of IDHmut patients [14, 16]. To our knowledge, this is the largest series of lower-grade gliomas comprehensively analyzing the prognostic relevance of reoperation versus non-surgical treatment. Despite different proportions of EOR categories and adjuvant treatment at 1^st^ diagnosis, treatment groups were balanced for most confounders at recurrence, including functional status, glioma subtype and WHO grade, and imbalances (sex, localization) were incorporated in the multivariable model. Lead-time bias was mitigated by adjusting for the interval between recurrence imaging and treatment start. Nevertheless, given the long observation period and the retrospective study design, treatment decisions, particularly regarding re-resection, were not standardized and inherently introduce selection bias. Tumor volume, an established prognostic factor, was not assessed in non-surgical cases, raising the possibility that tumor size influenced treatment selection. Treatment heterogeneity, influenced by changing therapeutic paradigms, represents another source of bias. Yet, similar proportions of patients were treated across pre-specified eras, starting treatment at 1^st^ recurrence mostly between 2011 and 2021, often guided by multidisciplinary tumor board recommendations.

This study investigated whether resection at 1^st^ recurrence was associated with improved survival. Notably, 18 patients (18.4%) of the non-surgical cohort underwent repeat resection at a later timepoint, and patients ever undergoing re-resection (*n* = 68) showed a trend towards prolonged SAR. However, the primary objective was to evaluate the effect of re-resection at the earliest possible recurrence. Because treatment burden increases with each additional recurrence, prolonging PFS-2 becomes an important therapeutic goal.

In multivariable analysis, symptomatic recurrence predicted poor outcome, irrespective of treatment group. This emphasizes the need to define the optimal timing of re-interventions, particularly reoperation, balancing the individual functional neuroplasticity against continuous tumor growth and invasion of eloquent areas [24]. In newly diagnosed IDHmut glioma, early surgery is beneficial [9, 25]. Given the influence of preoperative TVs on resectability, early repeat resection, ideally before symptom onset, should be considered in recurrent glioma as well.

Beyond outcome, reoperation enables assessment of malignant transformation. In our surgical cohort, this was observed in 10%, lower than previously reported for WHO grade 2 gliomas (19–74% [24]), likely reflecting exclusion of astrocytoma with CDKN2A/B homozygous deletion [2, 19]. In the non-surgical cohort, malignant transformation was not assessed, since neither re-biopsy nor advanced molecular imaging was routinely performed. Nonetheless, the clinical relevance is limited because recurrence usually leads to treatment irrespective of WHO grade [3].

Eloquent location was noted in similar proportions of surgical (30%) and non-surgical (36.7%) patients, with comparable functional status (KPS, NANO, symptomatic recurrence). Making use of surgical adjuncts (iMRI 94%; awake surgery 16%; IONM 12%) also in patients with incomplete resections, the rate of permanent neurological deficits after reoperation was low (2%), consistent with prior studies (0–8.5% [24]). Accordingly, functional status (KPS, NANO) remained stable at a high level at 7 days and 3 months after surgery. This aligns with previous reports that multiple surgeries can be safely performed under function-based resection guidance [26–29]. Surgical complications requiring revision were rare (4%), reflecting the generally low treatment intensity in lower-grade glioma as a predisposing factor. Thus, repeat resection was associated with prolonged PFS-2 at acceptable risk, supporting its role in the multimodal treatment of recurrent lower-grade glioma.

Strengths of this study include the long follow-up (median 11.7 years), molecular characterization according to WHO 2016 or 2021, exclusion of WHO grade 4 tumors and astrocytomas with homozygous CDKN2A/B deletion and volumetric assessment of EOR. Limitations include the moderate sample size of the surgical cohort, which precluded the identification of prognostic resection thresholds, lack of volumetric data in non-surgical cases, and the retrospective design, with treatment decisions based on the neuropathological diagnosis and treatment options available at the time of treatment, spanning three decades. Furthermore, the number of events - particularly for SAR and in surgical patients - was limited, making data maturation an important constraint. Because lower-grade glioma patients experience long-term survival, randomized trials comparing surgical vs. non-surgical treatment at recurrence seem neither feasible nor justifiable, given the emerging evidence favoring re-resection.

## Conclusion

In this single-center observational cohort of IDHmut lower-grade glioma, resection at 1^st^ recurrence was safe and associated with prolonged PFS-2 compared to non-surgical treatment, especially when GTR was achieved. However, treatment heterogeneity and selection biases inherent to the study design underscore the need for validation in larger, multicenter, era-harmonized cohorts incorporating comprehensive molecular profiling, standardized RANO endpoints and volumetric assessment. Since smaller preoperative tumor volumes favor resectability, further work should also address the optimal timing of repeat surgery, balancing tumor growth with individual functional neuroplasticity.

## supplementary material

Below is the link to the electronic supplementary material.

Supplementary Material 1

Supplementary Material 2

Supplementary Material 3

Supplementary Material 4

## Data Availability

The dataset generated and analyzed during the current study is available from the corresponding author on reasonable request.

## Declarations

### Standard of reporting

This observational study was performed in adherence with the STROBE guidelines. See **Supplementary Table** 2 for the STROBE checklist.
