# Supplementary material for: Prognostic relevance of resection at first recurrence in isocitrate dehydrogenase mutant lower-grade glioma: results from a retrospective, single-center, volumetric analysis
Source: J Neurooncol. 2026 Mar 11;177(1):42. doi: 10.1007/s11060-025-05353-x (PMC12979295; doi:10.1007/s11060-025-05353-x)
Supplement: Supplementary file 1 — Supplementary Material 1 [file 11060_2025_5353_MOESM1_ESM.pptx]

## Slide 1
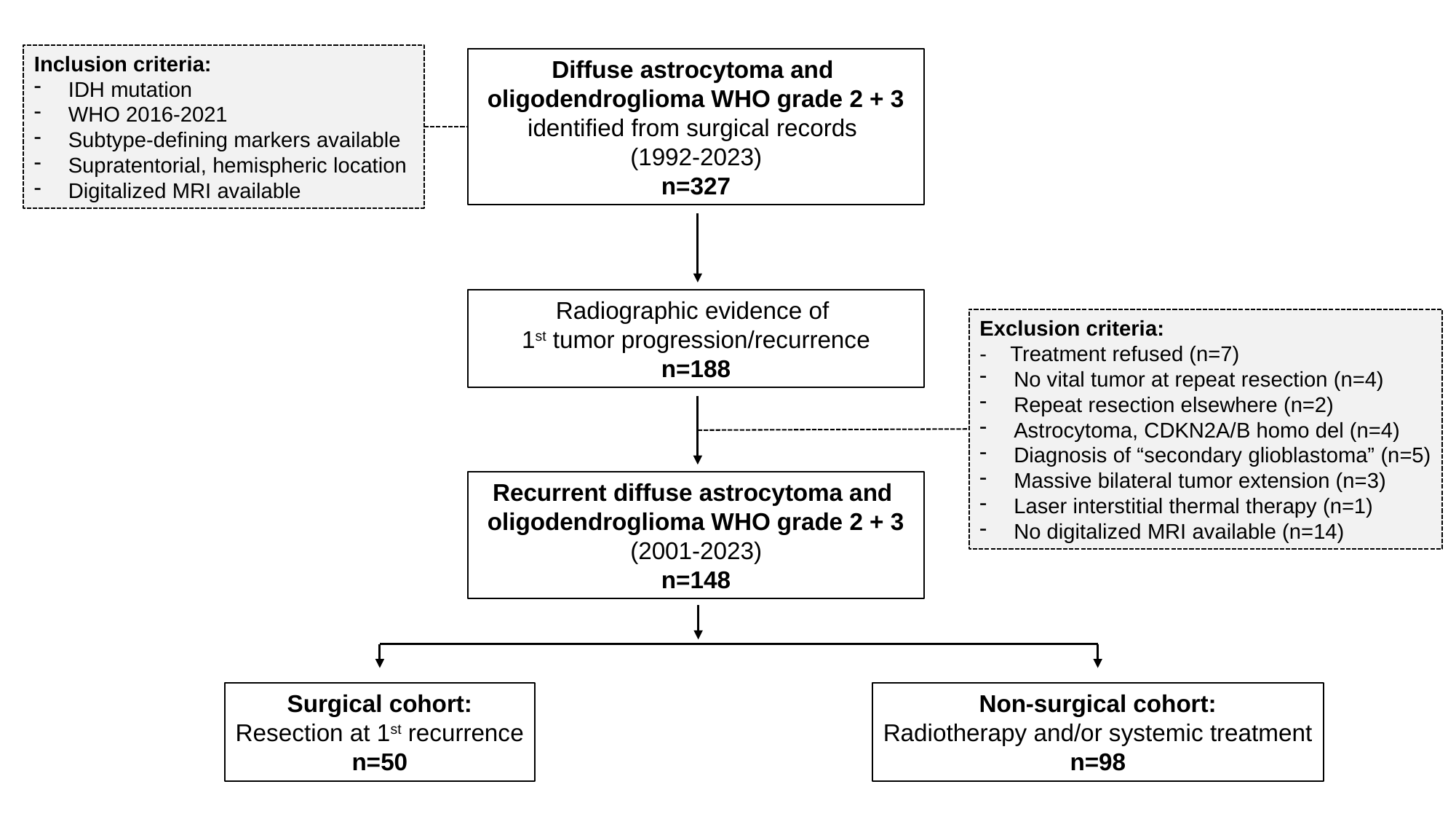

Inclusion criteria:
IDH mutation
WHO 2016-2021
Subtype-defining markers available
Supratentorial, hemispheric location
Digitalized MRI available
Diffuse astrocytoma and
oligodendroglioma WHO grade 2 + 3
identified from surgical records
(1992-2023)
n=327
Radiographic evidence of
1st tumor progression/recurrence
n=188
Exclusion criteria:
- Treatment refused (n=7)
No vital tumor at repeat resection (n=4)
Repeat resection elsewhere (n=2)
Astrocytoma, CDKN2A/B homo del (n=4)
Diagnosis of “secondary glioblastoma” (n=5)
Massive bilateral tumor extension (n=3)
Laser interstitial thermal therapy (n=1)
No digitalized MRI available (n=14)
Recurrent diffuse astrocytoma and
oligodendroglioma WHO grade 2 + 3
(2001-2023)
n=148
Surgical cohort:
Resection at 1st recurrence
n=50
Non-surgical cohort:
Radiotherapy and/or systemic treatment
n=98
