# Supplementary figures and images for: Prognostic relevance of resection at first recurrence in isocitrate dehydrogenase mutant lower-grade glioma: results from a retrospective, single-center, volumetric analysis
Source: J Neurooncol. 2026 Mar 11;177(1):42. doi: 10.1007/s11060-025-05353-x (PMC12979295; doi:10.1007/s11060-025-05353-x)

## Slide 1
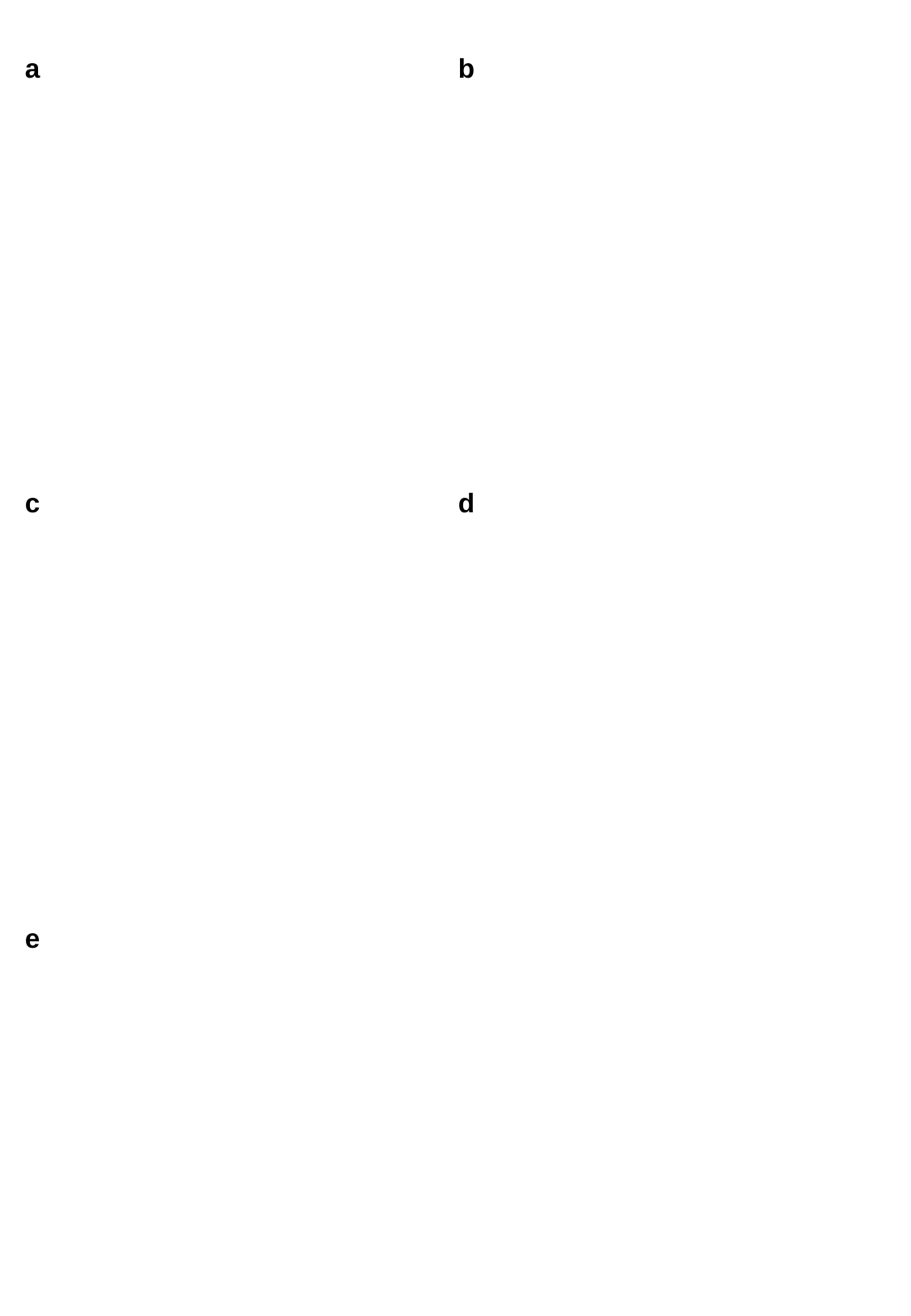

a
b
c
d
e

Supplement: Supplementary file 2 — Supplementary Material 2 [file 11060_2025_5353_MOESM2_ESM.pptx]
