# Supplementary material for: Prognostic relevance of resection at first recurrence in isocitrate dehydrogenase mutant lower-grade glioma: results from a retrospective, single-center, volumetric analysis
Source: J Neurooncol. 2026 Mar 11;177(1):42. doi: 10.1007/s11060-025-05353-x (PMC12979295; doi:10.1007/s11060-025-05353-x)
Supplement: Supplementary file 3 — Supplementary Material 3 [file 11060_2025_5353_MOESM3_ESM.docx]

**Suppl. Table 1 Univariate analysis of progression-free survival 2 and survival after recurrence in patients with recurrent IDHmut lower-grade glioma** (n=148)

| **Variable** | **PFS-2** | **SAR** |
| --- | --- | --- |
| Sex | 0.066 | 0.454 |
| **At 1^st^ recurrence** |  |  |
| Age (median; 42 years) | 0.992 | 0.574 |
| KPS ≥ 90 | 0.218 | **<0.001** |
| NANO = 0 | 0.126 | **0.005** |
| Seizures | **0.036** | 0.715 |
| Symptomatic recurrence | **<0.001** | **<0.001** |
| Tumor lateralization (right vs. left) | 0.755 | **0.016** |
| Tumor localization (frontal vs. others) | 0.050 | **0.042** |
| Tumor eloquence | 0.281 | 0.095 |
| Repeat resection | **0.010** | 0.058 |
| Histology | 0.232 | **0.002** |
| WHO grade | **0.041** | **<0.001** |

**Suppl. Table 1 Univariate analysis of progression-free survival 2 and survival after recurrence in patients with recurrent IDHmut lower-grade glioma** (n=148)

P-values were assessed by Log-rank tests and given in bold if significance level (≤ 0.05) was reached.

PFS-2: progression-free survival 2; SAR: survival after recurrence
