# Supplementary material for: Prognostic relevance of resection at first recurrence in isocitrate dehydrogenase mutant lower-grade glioma: results from a retrospective, single-center, volumetric analysis
Source: J Neurooncol. 2026 Mar 11;177(1):42. doi: 10.1007/s11060-025-05353-x (PMC12979295; doi:10.1007/s11060-025-05353-x)
Supplement: Supplementary file 4 — Supplementary Material 4 [file 11060_2025_5353_MOESM4_ESM.pdf]

STROBE Statement—checklist of items that should be included in reports of observational studies

	Item No.	Recommendation	Page No.	Relevant text from manuscript
**Title and abstract**	1	(*a*) Indicate the study's design with a commonly used term in the title or the abstract	Title:	"Results from a Retrospective, Single-Center, Volumetric Analysis"
			Abstract:	"This observational single-center study…" "We retrospectively analyzed…"
		(*b*) Provide in the abstract an informative and balanced summary of what was done and what was found		Abstract provided
**Introduction**				
Background/rationale	2	Explain the scientific background and rationale for the investigation being reported		e.g. "…a potential survival benefit (of repeat resection) is still debated…"
Objectives	3	State specific objectives, including any prespecified hypotheses		"Hence, we investigated whether resection at 1st recurrence – especially gross total resection (GTR) - compared to non-surgical treatment is associated with progression-free survival after recurrence (PFS-2) and survival after recurrence (SAR) (…). We also evaluated surgical complications and functional outcomes associated with repeat resection."
**Methods**				

Study design	4	Present key elements of study design early in the paper	see Title, Abstract and Patient Sample paragraph
Setting	5	Describe the setting, locations, and relevant dates, including periods of recruitment, exposure, follow-up, and data collection	Patient Sample paragraph ("treated between 1992 and 2023"; "treatment start at 1st recurrence across different study eras"; "follow-up until March 11th, 2025)
Participants	6	(*a*) *Cohort study*—Give the eligibility criteria, and the sources and methods of selection of participants. Describe methods of follow-up<br>*Case-control study*—Give the eligibility criteria, and the sources and methods of case ascertainment and control selection. Give the rationale for the choice of cases and controls<br>*Cross-sectional study*—Give the eligibility criteria, and the sources and methods of selection of participants	Patient Sample paragraph (detailed inclusion/exclusion criteria)<br>see flow chart (Suppl. Figure 1)
		(*b*) *Cohort study*—For matched studies, give matching criteria and number of exposed and unexposed<br>*Case-control study*—For matched studies, give matching criteria and the number of controls per case	Not applicable.
Variables	7	Clearly define all outcomes, exposures, predictors, potential confounders, and effect modifiers. Give diagnostic criteria, if applicable	Volumetric Analysis paragraph; Statistical Analysis paragraph
Data sources/ measurement	8*	For each variable of interest, give sources of data and details of methods of assessment (measurement). Describe comparability of assessment methods if there is more than one group	MRI protocol; segmentation method;
Bias	9	Describe any efforts to address potential sources of bias	Multivariable adjustment
Study size	10	Explain how the study size was arrived at	Patient Sample paragraph<br>see flow chart (Suppl. Figure 1)

Continued on next page

Quantitative variables	11	Explain how quantitative variables were handled in the analyses. If applicable, describe which groupings were chosen and why	Age at recurrence, Time from recurrence imaging to treatment start: continuous variable in multivariable Cox proportional hazards model
Statistical methods	12	(*a*) Describe all statistical methods, including those used to control for confounding	Statistical Analysis paragraph
		(*b*) Describe any methods used to examine subgroups and interactions	Stratification by treatment groups/residual tumor volumes/tumor subtypes
		(*c*) Explain how missing data were addressed	Only cases without missing variables entered multivariable models.
		(*d*) *Cohort study*—If applicable, explain how loss to follow-up was addressed<br>*Case-control study*—If applicable, explain how matching of cases and controls was addressed<br>*Cross-sectional study*—If applicable, describe analytical methods taking account of sampling strategy	Survival definitions provided (censored to last follow-up in case of lost to follow-up).
		(*e*) Describe any sensitivity analyses	Not performed.
**Results**			
Participants	13*	(a) Report numbers of individuals at each stage of study—eg numbers potentially eligible, examined for eligibility, confirmed eligible, included in the study, completing follow-up, and analysed	Patient Characteristics: n=148 patients included.<br>see flow chart (Suppl. Figure 1)
		(b) Give reasons for non-participation at each stage	see flow chart (Suppl. Figure 1)
		(c) Consider use of a flow diagram	see flow chart (Suppl. Figure 1)
Descriptive data	14*	(a) Give characteristics of study participants (eg demographic, clinical, social) and information on exposures and potential confounders	Patient Characteristics<br>Table 1; Table 3
		(b) Indicate number of participants with missing data for each variable of interest	CDKN2A/B status; volumetric data in non-surgical cohort
		(c) *Cohort study*—Summarise follow-up time (eg, average and total amount)	2nd paragraph: 140 months
Outcome data	15*	*Cohort study*—Report numbers of outcome events or summary measures over time	Table 1: deaths; 2nd progression; functional outcomes
		Case-control study—Report numbers in each exposure category, or summary measures of exposure	

		Cross-sectional study—Report numbers of outcome events or summary measures	
Main results	16	(*a*) Give unadjusted estimates and, if applicable, confounder-adjusted estimates and their precision (eg, 95% confidence interval). Make clear which confounders were adjusted for and why they were included	2nd paragraph: univariate log-rank tests and multivariable Cox proportional hazards analysis<br>Table 2<br>Suppl. Table 1
		(*b*) Report category boundaries when continuous variables were categorized	Quartiles of residual tumor volume
		(*c*) If relevant, consider translating estimates of relative risk into absolute risk for a meaningful time period	Not applicable.

Continued on next page

Other analyses	17	Report other analyses done—eg analyses of subgroups and interactions, and sensitivity analyses	GTR vs. residual tumor volume; comparison including non-surgical patients / “ever repeat resection” / stratification by residual tumor volume and tumor subtype
**Discussion**			
Key results	18	Summarise key results with reference to study objectives	First paragraph: “In this cohort of molecularly characterized recurrent lower-grade glioma, repeat resection at 1st recurrence was associated with prolonged PFS-2 (median 7.75 years) and low rates of neurological (2%) and surgical (4%) complications. Particularly, GTR prolonged PFS-2 compared to any incomplete resection or non-surgical treatment.”
Limitations	19	Discuss limitations of the study, taking into account sources of potential bias or imprecision. Discuss both direction and magnitude of any potential bias	Entire discussion (selection bias; lead-time bias; immortal-time bias; treatment heterogeneity; strengths & limitations)
Interpretation	20	Give a cautious overall interpretation of results considering objectives, limitations, multiplicity of analyses, results from similar studies, and other relevant evidence	Entire discussion (comparison with previous studies; discussion of selection bias; strengths & limitations)
Generalisability	21	Discuss the generalisability (external validity) of the study results	Final paragraph of discussion
**Other information**			
Funding	22	Give the source of funding and the role of the funders for the present study and, if applicable, for the original study on which the present article is based	Statements & Declarations: Funding

*Give information separately for cases and controls in case-control studies and, if applicable, for exposed and unexposed groups in cohort and cross-sectional studies.

Note: An Explanation and Elaboration article discusses each checklist item and gives methodological background and published examples of transparent reporting. The STROBE checklist is best used in conjunction with this article (freely available on the Web sites of PLoS Medicine at http://www.plosmedicine.org/, Annals of Internal Medicine at http://www.annals.org/, and Epidemiology at http://www.epidem.com/). Information on the STROBE Initiative is available at www.strobe-statement.org.
